# Pediatric Bipolar Disorder: Subtype Trend and Impact of Behavioral Comorbidities

**DOI:** 10.3390/jcm3010310

**Published:** 2014-03-20

**Authors:** Thiyagu Rajakannan, Julie M. Zito, Mehmet Burcu, Daniel J. Safer

**Affiliations:** 1 Department of Pharmaceutical Health Services Research, University of Maryland School of Pharmacy, Baltimore MD-21201, USA; E-Mails: trajakannan@rx.umaryland.edu (T.R.); mburc001@umaryland.edu (M.B.); 2 Departments of Psychiatry and Pediatrics, Johns Hopkins Medical Institutions, Baltimore MD-21287, USA; E-Mail: dsafer@jhmi.edu

**Keywords:** pediatric bipolar disorder, behavioral comorbidities, psychotropic medication, antipsychotics, stimulants, antidepressants

## Abstract

The diagnosis of pediatric bipolar disorder (PBD) has increased dramatically in community-treated youth in the past 20 years. No previous study has assessed the trend in PBD subtype diagnoses or the impact of clinician-reported behavioral comorbidities (BC) on psychotropic medication prescribing patterns. This study aims: (1) to characterize national trends in PBD visits in relation to PBD subtypes; and (2) to assess differences in socio-demographic PBD subtype diagnostic patterns and psychotropic medications prescribed in PBD visits with and without behavioral comorbidities (w/w/o BC). PBD visits for 1999–2010 from the National Ambulatory Medical Care Survey (NAMCS) data were assessed using population-weighted chi-square and logistic regression analyses. While PBD visit rates were stable across 12 years, the proportional shift of subtype diagnosis from Bipolar I (89.0%) in 1999–2002 to Bipolar Not Otherwise Specified (NOS) (74.1%) in 2007–2010 was notable. Compared with PBD without behavioral comorbidities (w/o BC), PBD visits w/BC had greater proportions of the bipolar-NOS subtype, more males, 2–14-year-olds, and more publicly-insured visits. The prescription of antipsychotics (60% *vs.* 61%) was common in PBD visits regardless of the presence of behavioral comorbidities. Stimulants were the predominant class prescribed for PBD visits with BC (67.8% *vs.* 9.4%). Antidepressants were significantly greater in PBD visits without BC (41.6% *vs.* 21.0%). Overall one-third of PBD youth visits were prescribed antipsychotics concomitant with other psychotropic classes. Behavioral conditions accompanying PBD visits were prominent, suggesting the need for monitoring and evaluating the outcomes of complex medication regimens in community populations.

## 1. Introduction

Pediatric bipolar disorder (PBD) is a serious mental disorder that can lead to disruption in the lives of children and adolescents [1]. Bipolar disorder, once considered rare in adolescents and younger children, has been increasingly diagnosed in community populations over the last decade [2,3]. Blader and Carlson [2] reported population-adjusted rates of hospital discharges of children with a primary diagnosis of PBD that increased linearly over eight years. The U.S. rate between 1996 and 2004 grew 5.6-fold for children and four-fold for adolescents [2]. The study by Moreno *et al.* showed that the annual estimate of PBD office visits increased 40-fold from 1994–1995 to 2002–2003 [3]. This rapid increase of PBD diagnosis has raised concerns of the over-diagnosis of this disorder among children and adolescents [3,4]. 

The overlapping of symptoms, e.g., distractibility, pressured speech, and irritability, have made clinicians and researchers aware of the difficulty of diagnosing comorbid behavior conditions, like attention-deficit hyperactivity disorder (ADHD) in children with PBD [3,4,5,6]. Recently, Dusetzina *et al.* analyzed private insurance claims in 2007 for youth <18 years of age. Among 16,641 youth with clinician-reported PBD, approximately 30% had a comorbid diagnosis of ADHD [7]. The distribution of the PBD subtype was PBD-I/PBD-II/PBD-Not Otherwise Specified (NOS) 38.3%/11.2%/50.5%. Along with the PBD diagnostic growth, there has been a corresponding increase in medication treatment patterns, consistent with the expanded use of antipsychotics [8,9] and concomitant psychotropic class use regardless of diagnosis [10].

The high prevalence of PBD-NOS subtype raises the question about its diagnostic reliability. NOS diagnoses do not meet the defined criteria in the Diagnostic and Statistical Manual of Mental Disorders (DSM-IV) [11]. These diagnoses are primarily based on sub-threshold symptom levels. Additionally, increased comorbid conditions due to overlapping of symptoms can indicate greater severity and justify additional medications, which can lead to increased risks associated with drug combinations [12].

Despite the high visibility of this topic, no information exists on the temporal trends in PBD subtypes, the impact of the presence of behavioral comorbidity in youth diagnosed with PBD and the change in psychotropic medications prescribed during these visits over 12 years. Therefore, this study aims: (a) to assess the time trends in PBD and its subtypes across 12 years from a national sample of physician office visit data; and (b) to assess the impact of the presence of behavioral comorbidities on PBD subtypes, patient visit characteristics and psychotropic medication prescription patterns. 

## 2. Methods

### 2.1. Data Source and Survey Design

Data were drawn from annual U.S. National Ambulatory Medical Care Surveys (NAMCS) for 1999–2010. The NAMCS is a federally sponsored survey conducted by the National Center for Health Statistics (NCHS). NAMCS data are based on a multistage probability sampling design collected from non-federally employed physicians engaged in direct patient care during a randomly assigned one-week reporting period [13]. During this period, data for a systematic random sample of visits are recorded by the physician or office staff on an encounter form provided for that purpose. Data are obtained on selected patients’ demographic characteristics; physician-reported diagnoses, prescribed medications and services provided. Following NCHS recommendations, NAMCS medical visit data from contiguous years are combined to produce stable estimates. In order to estimate the temporal trends of youth visits with a diagnosis of PBD, the data were grouped into three four-year periods as follows: 1999–2002, 2003–2006 and 2007–2010. Each visit is assigned a value, the sum of which projects to an estimate of the total medical visits nationally. This value is referred to as weighted value estimation (WVE). The weighting procedures produce essentially unbiased national estimates and are derived from the following components: (1) an inflated estimate based on national census; (2) adjustment for non-response; (3) a ratio adjustment to fixed totals; and (4) weight smoothing [14]. We included a weighted column percentage (WC%), 95% confidence interval (CI) of WVE along with the number (*N*) of unweighted visits. Estimates based on fewer than 30 visits are unreliable. There were 424 PBD visits identified from total visits (*N* = 47,386) for 2–19 years olds. Across the study years, survey response rates varied between 58.3% and 70.4%, with a median response rate of 62.5%. The ratio of physicians to reported visits was 1:22 for the study years. 

### 2.2. Study Variables

#### 2.2.1. Demographic, Visit and Prescribing Characteristics

For the present study, age was categorized as 2–9, 10–14 and 15–19 years. Race/ethnicity was categorized as white and non-white (African American, Hispanic, Native American, Pacific Islander and Asian or more than one race). Data regarding sources of payment for the visit were collapsed into 2 mutually exclusive categories: private insurance, including self-payment, and public insurance (Medicare, Medicaid, other government insurance, no charge and unknown payment source). Physician specialties were grouped as psychiatry and non-psychiatry (general practice, family practice, pediatrics, neurology and other specialties). Four regions were defined by U.S. census categories as Northeast, South, Midwest and West-Pacific.

#### 2.2.2. Diagnosis

Office visit psychiatric diagnoses were recorded by treating physicians according to the International Classification of Diseases, Ninth Revision, Clinical Modification (ICD-9-CM) codes. The visits were classified as bipolar mania (296.0, 296.1 and 296.4), bipolar depression (296.5), bipolar mixed (296.6) and bipolar episode unspecified (296.7). Subtype groupings included the following: Bipolar I (296.0, 296.1, 296.4, 296.5, 296.6, 296.7), Bipolar II (296.89) and Bipolar-NOS (296.80).

Psychiatric comorbidities were identified for PBD visits that had additional clinician-reported codes of mental disorders. Comorbid psychiatric diagnoses were categorized as: 314–314.99 for attention deficit hyperactivity disorder (ADHD); 312.00–312.49 and 312.80–312.99 for conduct disorder (CD); 313.81 for oppositional defiant disorder (ODD); 296.2–296.3, 300.4, 311 for depression; 300–300.3, 300.5–300.9 and 309.81 for anxiety disorders. All other ICD-9-CM codes between 290 and 319, excluding the above-mentioned categories, were labeled as “other psychiatric disorder”. Up to three diagnoses could be recorded for each pediatric bipolar disorder visit. Patient visits were categorized as PBD with behavior disorder (with co-morbid diagnosis of ADHD, CD or ODD) and without behavior disorder (PBD with other psychiatric comorbid conditions) for the analysis of PBD w/w/o BC.

#### 2.2.3. Psychotropic Medications

Psychotropic medications prescribed for the treatment of PBD include seven classes: lithium, antipsychotics (atypical and conventional antipsychotics (ATP)), anticonvulsant-mood stabilizers (ATC-MS), antidepressants (ATD), anti-anxiety/hypnotics, alpha-agonists and stimulants. Additionally, they were categorized as monotherapy or ATP-concomitant regimens. ATC-MS included carbamazepine, oxcarbazepine, divalproex, valproic acid, lamotrigine and topiramate. PBD visits with prescribed psychotropic medication classes were population weighted and reported as column percentages with a 95% confidence interval (CI). Furthermore, the most frequently prescribed ATP regimens concomitant with other psychotropic classes were reported as column percentages. 

### 2.3. Analytical Plan

Population-weighted trends in PBD visits were assessed for 1999 to 2002, 2003 to 2006 and 2007 to 2010 using the chi-square statistic. Likewise, in the same time-periods, proportional differences in PBD subtypes were assessed. Subsequently, to study the impact of behavioral comorbidities on PBD visits, we combined the most recent years (2003–2010) to improve statistical reliability. In this analysis, population weighted differences in socio-demographic, clinical, administrative and prescribed psychotropic medication class characteristics of PBD visits with and without behavioral comorbidity were assessed with chi-square statistics. Population-weighted multivariable logistic regression modeling was employed to report the adjusted odds ratio (AOR) with 95% CI for PBD with behavioral comorbidity *vs*. PBD w/o behavioral comorbidity, adjusting for age group, gender, race/ethnicity, payment type, region and PBD subtype. SAS version 9.2 (SAS Institute, Inc., North Carolina, Cary) was used for all analyses in this study. 

## 3. Results

### 3.1. Recent Trends in PBD Office Visits, PBD Subtypes and Antipsychotic Medication Regimens

**Figure 1:**
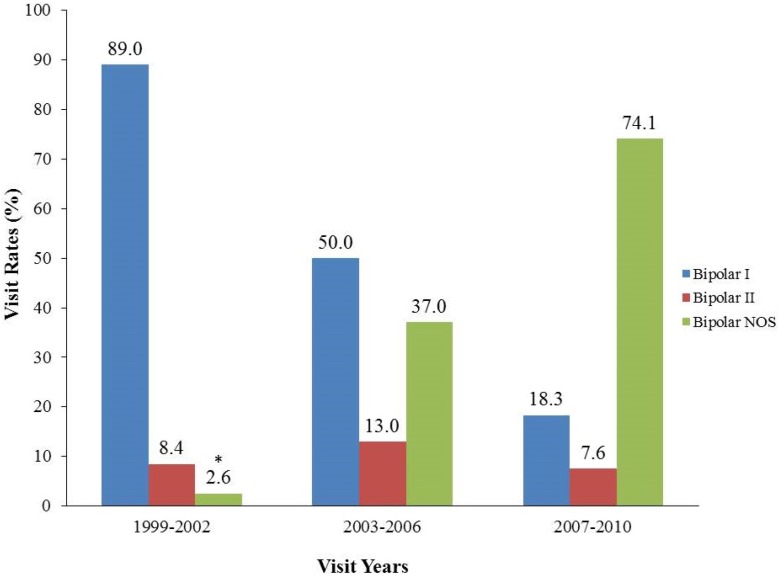
Proportional distribution of pediatric bipolar disorder (PBD) visits according to subtype diagnoses in three time periods ^a^.

^a^ Data are from the National Ambulatory Medical Care Survey; NOS, not otherwise specified; * Represents unreliable estimates, due to a small sample size.

The percentage of PBD visits as a proportion of total pediatric visits remained stable among youths in recent study years: 0.4% (95% CI, 0.2–0.6) in 1999–2002 and 0.4% (95% CI, 0.3–0.5) in 2007–2010 (data not shown). By contrast, there was a proportional shift of subtype diagnosis from PBD-I (89.0%) in 1999–2002 to PBD-NOS (74.1%) in 2007–2010 (χ^2^ = 60.3, df = 4, *p* < 0.0001) (Figure 1). Specifically, PBD-I decreased from 89.0% to 18.3%, whereas PBD-NOS significantly increased from 2.6% to 74.1%. In the most recent period, 2007–2010, the PBD-I/PBD-II/PBD-NOS distribution was 18.3%/7.6%/74.1%, showing the great preponderance of NOS in recent years. 

Table 1 shows the demographic, clinical and administrative characteristics of PBD youth visits with and without a behavior disorder for the eight-year period, 2003**–**2010. Compared to PBD visits without behavioral comorbidity, PBD visits with behavioral comorbidities represented greater proportions of the Bipolar-NOS subtype (60.8% *vs.* 45.3%), as well as more males (69.7% *vs.* 46.5%), 2**–**14-year-olds (65.2% *vs.* 31.5%) and publicly-insured youth (55.0% *vs.* 40.0%). Regardless of comorbid behavioral status, more than 75% of PBD visits were made to psychiatrists. The prescription of antipsychotics was common (60.0% *vs.* 61.0%) in PBD visits regardless of the presence of comorbid behavior disorders. While stimulants were the predominant prescribed class for PBD visits with comorbid behavioral disorder (67.8% *vs.* 9.4%), antidepressants were significantly greater in PBD visits without comorbid behavioral disorders (20.9% *vs.* 41.6%). 

Table 2 illustrates that antipsychotic monotherapy was more common among PBD without behavioral comorbidity. More specifically, ATP with concomitant stimulants was the leading regimen for PBD with behavioral comorbidities compared with PBD without behavioral comorbidities (*p* < 0.0001). Overall one-third or more of PBD visits had antipsychotic (ATP) regimens with one or more concomitant psychotropic classes (data not shown). The use of ATP with anticonvulsant-mood stabilizers or antidepressants did not differ according to behavioral comorbid status.

**Table 1:** Demographics, clinical characteristics and prescribed psychotropic medication classes in office-based PBD visits with and without behavioral comorbidities for 2003–2010. ^a^ *N* = 318.

Characteristic	PBD with behavioral comorbidities		PBD without behavioral comorbidities		
	*N*	WC%	*N*	WC%	*p* Value
**Total**	162		156		
Bipolar NOS	96	60.8	68	45.3	0.02
Bipolar I & II	66	39.2	88	54.7	
**Gender**					
Male	119	69.7	73	46.5	<0.001
Female	43	30.3	83	53.5	
**Age group, years**					
2–9	36	19.1	13	7.5	<0.0001
10–14	70	46.1	39	24.0	
15–19	56	34.8	104	68.5	
**Race/Ethnicity**					
White	131	82.9	126	81.6	NS
Non-White	31	17.2	30	18.4	
**Payment type**					
Private	71	45.0	91	60.5	0.03
Public	91	55.0	65	39.6	
**Type of Practice**					
Psychiatry	148	85.1	132	75.3	NS
Non-Psychiatry	14 ^†^	14.9	24 ^†^	24.7	
**Region**					
North-East	23 ^†^	13.6	37	12.2	NS
Mid-West	54	27.9	35	13.1	
South	38	30.9	36	13.8	
West	47	27.6	48	14.8	
**Prescribed psychotropic medications**					
Any psychotropic visit	151	94.6	147	92.8	NS
Antipsychotics	105	59.6	97	61.3	NS
Antidepressants	39	21.0	65	41.6	0.001
Anxiolytics & Hypnotics	6 ^†^	3.4	15 ^†^	11.5	0.01
Lithium	17 ^†^	9.7	17 ^†^	9.7	NS
Alpha-agonist	16 ^†^	8.3	5 ^†^	3.6	NS
Anticonvulsant	68	44.7	66	40.2	NS
Stimulants	104	67.8	14 ^†^	9.4	<0.0001

^a^ Data are from the National Ambulatory Medical Care Survey; PBD, pediatric bipolar disorder; ^†^ Represents unreliable estimates, due to a small sample size; *N*, number; WC%, weighted column percentage; NS, not significant.

**Table 2:** Antipsychotic (ATP) drug regimens prescribed for PBD with and without behavioral comorbidities during 2003–2010; *N* = 318.

	PBD with behavioral comorbidities		PBD without behavioral comorbidities		
ATP regimens	*N*	WC%	*N*	WC%	*p* Value
ATP monotherapy	10 ^†^	4.2	29 ^†^	19.7	<0.001
ATP + ≥1 concomitant psychotropic classes	95	41.2	68	34.9	
ATP + concomitant stimulant	72	19.5	8 ^†^	2.7	<0.0001
ATP + concomitant ATC-MS	38	10.4	38	12.2	NS
ATP + concomitant ATD	28 ^†^	7.2	36	12.2	NS

PBD, pediatric bipolar disorder; *N*, number; WC%, weighted column percentage; ATP, antipsychotics; ATC-MS, anticonvulsant-mood stabilizers; ATD, antidepressants; ^†^ Represents unreliable estimates due to small sample sizes; NS, not significant.

### 3.2. Multivariable Analyses

Table 3 displays the results of the logistic regression analysis showing that the adjusted odds of having a behavioral comorbidity was significantly (AOR = 2.3 (95% CI, 1.2, 4.0)) greater in Bipolar-NOS than in the reference group, Bipolar I and II. The adjusted odds of having PBD with behavioral comorbidities was 5.3 (95% CI, 2.7, 10.6) times greater in 2–9-year-olds compared with older youth; and 2.3 (95% CI, 1.3, 4.0) times greater among males. 

In a separate model (data not shown), PBD visits with behavior disorders had 21 times (AOR = 21.0 (95% CI, 10.1, 43.3)) greater adjusted odds of having prescribed stimulants.

**Table 3:** Adjusted odds ratios (AOR) of behavioral comorbidity *vs.* no behavioral comorbidity in PBD visits.

Variable	AOR	95% CI
**Diagnosis (reference: Bipolar I & II)**		
Bipolar NOS	2.3	1.3–4.1
**Gender (reference: female)**		
Male	2.3	1.3–4.0
**Age group (reference: 15–19 years)**		
2–9 years	5.3	2.7–10.6
10–14 years	3.7	1.8–7.4
**Race-ethnicity (reference: white)**		
Non white	0.6	0.3–1.3
**Payment (reference: public)**		
Private	0.6	0.3–1.0

AOR, adjusted odds ratio (adjusted for age group, gender, race/ethnicity, payment type, region and PBD subtypes); CI, confidence interval; NOS, not otherwise specified.

## 4. Discussion

There are three major findings from this study of pediatric bipolar disorder (PBD) in community treated populations across the United States. The first documents the dramatic change in the medical visit PBD diagnosis by subtype from 1999–2002 to 2007–2010. It is not unusual or unexpected that the largest PBD subtype would be “Not Otherwise Specified” (NOS), since this is the case in the epidemiologic survey research of adolescents [15] and clinical pediatric psychiatry assessments [16,17]. However, this was not the case in earlier national medical visit data from 1999–2002, when 89% of such visits were associated with a diagnosis of PBD-I, manic or mixed manic type. By contrast, over the next eight years, the diagnosis of PBD-I fell to 18.3%, while PBD-NOS rose dramatically from 2.6% to 74.0% of the total (Figure 1). 

PBD-NOS is diagnosed when the number of reported bipolar features are subthreshold (below the cut-off number required for a diagnosis) or when the duration of the bipolar episode is below the length of time criterion defined by the DSM. Stringaris and colleagues [18] using data from a national survey of youth in the UK evaluated those with manic-like episodes that met impairment criteria, but whose episode length was below the diagnostic threshold. The resultant findings were that only a few youth met the criteria for BP-I/BP-II and that the number who met diagnostic criteria for BP-NOS was 10 times that number. The authors concluded that youth diagnosed with BP-NOS are in all likelihood a distinct entity separate from youth meeting the full criteria for bipolar disorder.

The relative increase in PBD-NOS likely reflects shifting attitudes about the appropriateness of PBD-I or PBD-II for chronic disruptive behavior problems characterized by hyperactivity and irritability [19,20]. PBD-NOS may become the default diagnosis when ADHD severity, particularly aggression, is a major problem [21]. Among newly diagnosed PBD youth, one-third had a prior behavior diagnosis [22], further supporting the close relationship of PBD and behavior disorders. Finally, experts have debated whether the labeling as PBD symptoms could more properly be identified as severe disruptive behavior disorder [23]; the DSM-5 reflects this perspective in its promulgation of disruptive mood dysregulation disorder, which was initially referred to as temper dysregulation disorder [24].

The second major finding is that behavior disorders (*i.e.*, ADHD, ODD and CD) are frequently comorbid with PBD and that those with, compared with those without this comorbidity are significantly more often male, preadolescent and are diagnosed with the PBD-NOS subtype (Table 2). PBD with comorbid ADHD has been analyzed by Kent and Craddock in terms of the overlapping of some ADHD and manic-like symptoms in the DSM-5 and ICD-10 [5] and by Pataki and Carlson in DSM-5 [25]. These analyses are consistent with earlier cohort studies in Italy [26], the UK [18] and in U.S. adolescent PBD hospital admissions [27]. 

The third finding of note relates to the complexity of the prescribed medication regimens, which has been recognized as challenging [28]. PBD in community treatment is reported to involve 3.4 concomitant medications on average [29]. In the current study, antipsychotic medications had a similar prescribing rate for PBD youth with and without behavioral comorbidities (60% *vs.* 61%), but their combination with other classes, e.g., stimulants and anticonvulsant-mood stabilizers, raises questions. Concomitant use of antipsychotic and anticonvulsants is common [29,30], despite the mixed evidence of anticonvulsant efficacy, safety and tolerability in either short-term or long-term pediatric use [31], although the point has been disputed [32]. Perhaps single drug clinical trial comparisons to placebo are sufficient to justify monotherapy, but are they adequate to support combinations? Research on the use of anticonvulsants in PBD in prepubertal children has not been encouraging, due to the failure to enroll or sustain participation [33]. 

In addition, West *et al.* found a differential benefit for risperidone compared with divalproex in 8–18-year-olds diagnosed with PBD with comorbid behavioral symptoms, particularly aggression [34]. The potential pharmacologic interaction of dopamine agonists (stimulants) and dopamine blockers (antipsychotics) warrants greater understanding in terms of short-term and long-term effectiveness, safety and tolerability [35]. Close monitoring of complex multidrug regimens is recommended by the American Academy of Child and Adolescent Psychiatry (AACAP) practice parameter for pediatric bipolar disorder [36], particularly where atypical antipsychotics are combined with other potent classes [37].

### 4.1. Limitations

This study has several limitations. First, NAMCS survey analyses are based on outpatient physician visits rather than on patients as the unit of analysis. While a 1:1 relationship of persons to visits is not possible, trend analyses across time are accurate. Second, diagnoses in the NAMCS are based on the judgment of treating clinicians rather than on a research-level assessment. Third, because the surveys are cross-sectional and cover a limited timeframe, no information is available concerning the duration of medication use. Fourth, different phases of bipolar disorder (euthymic, manic, hypomanic, depressed) were not available as variables. Finally, a major issue of this NAMCS study relates to the modest statistical power of the individual NAMCS survey years to assess PBD, a relatively rare pediatric condition. To overcome this problem, the data were grouped as four-year periods for trends and as the most recent eight years from 2003 to 2010 for PBD w/w/o BC. Due to the low visit number, we could not distinguish between combinations of psychotropic medications prescribed for PBD with and without behavioral comorbidities. Potential interactions among the study variables are not explored due to the limited sample size. The value of NAMCS is its sophisticated sampling design, reporting of diagnostic and treatment information by health professionals and national scope across many years. 

### 4.2. Future Research and Practice Directions

Predictions of fewer psychiatric drugs in the pipeline and the waning interest of the pharmaceutical industry in psychiatric drug development [38] may energize clinical research efforts to create the infrastructure and methodology for robust outcomes research. Despite the calls for outcomes research, physicians are trained largely in an individual person model. Nevertheless, that model can be supplemented with training in a population-based model. In effect, this approach would extend beyond clinical trials into post-marketing surveillance, *i.e.*, outcomes research. Along with emerging electronic medical record capacity, brief computerized records of parent-recorded perspectives could accompany physician assessment of the outcome using epidemiologic methods to monitor individuals, as well as prospective cohorts. Federal research calls for proposals from the relatively new Patient-Centered Outcomes Research Center (PCORI) feature patient (or family) perspectives in treatment research and aim to reduce the gaps in existing knowledge of PBD [39]. Child psychiatry training programs can engage their trainees to use brief, patient-oriented monitoring systems to include the parent assessment of symptoms, functioning and adverse events in models that are practical and can be incorporated in community practice settings, despite their time constraints [40].

## 5. Conclusions

Pediatric bipolar disorder treatment in community-treated populations would benefit from clinical monitoring and evaluation studies of outcomes to better understand the shift to a preponderance of the PBD-NOS subtype and the prominent occurrence of PBD with behavioral comorbidity. The accompanying complexity in drug combinations, e.g., antipsychotics and stimulants, that is shown here demands the evaluation of benefits, safety and tolerability.
